# A human pluripotent stem cell model of catecholaminergic polymorphic ventricular tachycardia recapitulates patient-specific drug responses

**DOI:** 10.1242/dmm.026823

**Published:** 2016-09-01

**Authors:** Marcela K. Preininger, Rajneesh Jha, Joshua T. Maxwell, Qingling Wu, Monalisa Singh, Bo Wang, Aarti Dalal, Zachary T. Mceachin, Wilfried Rossoll, Chadwick M. Hales, Peter S. Fischbach, Mary B. Wagner, Chunhui Xu

**Affiliations:** 1 Division of Pediatric Cardiology, Department of Pediatrics, Emory University School of Medicine and Children's Healthcare of Atlanta, Atlanta, GA 30322, USA; 2 Wallace H. Coulter Department of Biomedical Engineering, Georgia Institute of Technology and Emory University, Atlanta, GA 30322, USA; 3 Department of Cell Biology, Emory University School of Medicine, Atlanta, GA 30322, USA; 4 Laboratory of Translational Cell Biology, Emory University School of Medicine, Atlanta, GA 30322, USA; 5 Department of Neurology, Emory University School of Medicine, Atlanta, GA 30322, USA

**Keywords:** Arrhythmia models, Cardiomyocytes, Ca^2+^ handling, CPVT, iPSCs

## Abstract

Although β-blockers can be used to eliminate stress-induced ventricular arrhythmias in patients with catecholaminergic polymorphic ventricular tachycardia (CPVT), this treatment is unsuccessful in ∼25% of cases. Induced pluripotent stem cell-derived cardiomyocytes (iPSC-CMs) generated from these patients have potential for use in investigating the phenomenon, but it remains unknown whether they can recapitulate patient-specific drug responses to β-blockers. This study assessed whether the inadequacy of β-blocker therapy in an individual can be observed *in vitro* using patient-derived CPVT iPSC-CMs. An individual with CPVT harboring a novel mutation in the type 2 cardiac ryanodine receptor (RyR2) was identified whose persistent ventricular arrhythmias during β-blockade with nadolol were abolished during flecainide treatment. iPSC-CMs generated from this patient and two control individuals expressed comparable levels of excitation-contraction genes, but assessment of the sarcoplasmic reticulum Ca^2+^ leak and load relationship revealed intracellular Ca^2+^ homeostasis was altered in the CPVT iPSC-CMs. β-adrenergic stimulation potentiated spontaneous Ca^2+^ waves and unduly frequent, large and prolonged Ca^2+^ sparks in CPVT compared with control iPSC-CMs, validating the disease phenotype. Pursuant to the patient's *in vivo* responses, nadolol treatment during β-adrenergic stimulation achieved negligible reduction of Ca^2+^ wave frequency and failed to rescue Ca^2+^ spark defects in CPVT iPSC-CMs. In contrast, flecainide reduced both frequency and amplitude of Ca^2+^ waves and restored the frequency, width and duration of Ca^2+^ sparks to baseline levels. By recapitulating the improved response of an individual with CPVT to flecainide compared with β-blocker therapy *in vitro*, these data provide new evidence that iPSC-CMs can capture basic components of patient-specific drug responses.

## Abstract (precis)

**Editors' choice:** Clinically observed drug response differentials to β-blocker and flecainide treatment in catecholaminergic polymorphic ventricular tachycardia can be modeled *in vitro* using patient-derived iPSC-cardiomyocytes.

## INTRODUCTION

Catecholaminergic polymorphic ventricular tachycardia (CPVT) is a life-threatening inherited arrhythmia that predisposes young individuals with structurally normal hearts to cardiac arrest. The autosomal dominant form of CPVT is linked to mutations in the gene encoding the type 2 ryanodine receptor (RyR2) (Priori et al., 2001), an ion channel responsible for the coordinated release of intracellular Ca^2+^ from the sarcoplasmic reticulum (SR) to the cytosol during systole. Evidence suggests that the majority of RyR2 mutations promote catecholamine-driven spontaneous Ca^2+^ release from the SR during diastole by reducing the threshold for store overload-induced Ca^2+^ release (Jiang et al., 2005). In turn, these diastolic Ca^2+^-release events generate an electrogenic, depolarizing transient inward current that leads to delayed afterdepolarizations (DADs) and triggered arrhythmias in CPVT (Paavola et al., 2007).

As catecholaminergic stress is key in eliciting CPVT symptoms, drugs that impede the action of endogenous catecholamines by blocking β-adrenergic receptors (β-AR) are the foundation of pharmacological CPVT therapy. However, for unknown reasons, ∼25% of CPVT patients are inadequately protected by β-blockers (Roston et al., 2015; Smith and MacQuaide, 2015). In the event that ventricular ectopy persists under β-blockade, the sodium channel blocker flecainide has emerged as an effective secondary agent for suppressing arrhythmias in CPVT (Watanabe et al., 2009; van der Werf et al., 2011). Ideally, clinicians would be able to anticipate an individual's receptivity to β-blocker therapy based on some molecular signature, and use the data to inform point-of-care treatment decisions. Patient-specific induced pluripotent stem cell (iPSC)-derived cardiomyocytes (CMs) offer an auspicious platform for achieving this aim (Sinnecker et al., 2013; Wilson and Wu, 2015). Although several reports have demonstrated pharmacological rescue of mutant RyR2 function in human CPVT iPSC-CMs (Itzhaki et al., 2012; Jung et al., 2012; Di Pasquale et al., 2013; Zhang et al., 2013), few studies directly correlate *in vitro* patient-specific drug response differentials to *in vivo* clinical data. A notable proof-of-principle study for this paradigm demonstrated that CPVT patient-derived iPSC-CMs can replicate *in vivo* individual drug responses to dantrolene in a mutation-specific manner (Penttinen et al., 2015). However, before patient-derived iPSC-CMs can be widely utilized for precision medicine, their capacity to model *in vivo* therapeutic idiosyncrasies must be comprehensively established.

The present study sought to determine whether a patient-specific response to therapeutic β-blockade can be observed *in vitro* in CPVT iPSC-CMs. To this end, iPSC lines were derived from an individual with CPVT harboring a novel RyR2*-*L3741P mutation whose ventricular ectopy was not abolished by the widely prescribed β-blocker nadolol, but was resolved with flecainide. As controls, iPSC lines were derived from two unrelated healthy individuals with no history of cardiac disease. iPSC-CMs differentiated from the two CPVT lines displayed Ca^2+^-handling defects generally associated with CPVT CMs, including increased spontaneous Ca^2+^ release during β-AR stimulation and reduced SR Ca^2+^ content compared with control cells. Consistent with the patient's *in vivo* outcomes, flecainide proved more effective than nadolol in reducing potentially arrhythmogenic Ca^2+^ release in iPSC-CMs derived from the individual during β-AR agonism. Further investigation of the therapeutic effects of flecainide on CPVT CMs following β-AR stimulation showed that it successfully improved Ca^2+^ homeostasis and mitigated electrical instability by reducing the incidence of DADs and asymmetrical beat periods.

These results support the hypothesis that iPSC-CMs can capture key components of patient-specific drug responses, and imply that CM-specific factors play a role in determining a patient's receptiveness to β-blocker therapy.

## RESULTS

### Flecainide preferentially resolves ventricular arrhythmias in CPVT patient

**Fig. 1.**
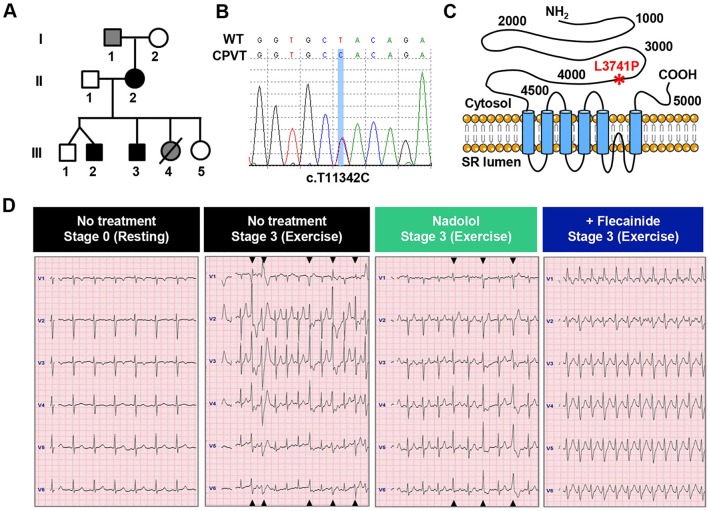
**Flecainide preferentially resolves ventricular arrhythmias in individual with CPVT.** (A) Pedigree of the subject (III-2). Black symbols, CPVT-affected individuals harboring the novel RyR2-L3741P mutation; gray symbols, CPVT-symptomatic with unverified genotype; open symbols, asymptomatic; line through symbol, deceased. (B) DNA sequencing of the patient reveals a T→C nucleotide substitution at position 11,342 in the coding sequence on chromosome 1 (i.e. c.T11342C). (C) Schematic representation of RyR2 channel and localization of the family's novel L3741P missense mutation (red asterisk). (D) The individual with CPVT's diagnostic exercise stress test (panels with black header) shows a normal basal electrocardiogram (ECG) and the development of polymorphic ventricular tachycardia during stage 3 exercise. Follow-up exercise stress tests reveal the persistence of multiform ventricular ectopy during stage 3 exercise despite β-blocker therapy with nadolol (panels with green header), but complete resolution of ventricular arrhythmias during stage 3 exercise with flecainide treatment (panels with blue header). Black triangles at the top and bottom of the ECG represent occurrences of ventricular ectopy observable across multiple electrodes.

The pedigree of the 12-year-old male individual with CPVT (III-2) selected for this study shows several affected family members demonstrating an autosomal dominant inheritance pattern of the syndrome (Fig. 1A). Genotyping of the individual, his brother and his mother identified a shared novel amino acid missense leucine→proline mutation at residue site 3741 in RyR2 (i.e. L3741P), caused by a T→C nucleotide substitution at position 11,342 in the coding sequence (i.e. c.T11342C) (Fig. 1B,C). The mutation is located outside the salient ‘hotspot’ regions where most RyR mutations cluster, which include regions in the N-terminal, central and C-terminal domains (Priori and Napolitano, 2005; Thomas et al., 2010). Echocardiography revealed a structurally normal heart (data not shown) and resting electrocardiogram was unremarkable (Fig. 1D). However, bicycle ergometer exercise stress testing evoked polymorphic ventricular tachycardia during stage 3 exercise at a peak heart rate of 167 bpm (Fig. 1D). The subject received an implantable cardiac defibrillator in addition to β-blocker treatment with nadolol (20 mg once daily; 0.74 mg/kg/day). A follow-up exercise stress test at nineteen months revealed that multiform ventricular arrhythmias persisted despite β-blockade (Fig. 1D), with ventricular ectopy starting during stage 1 exercise and progressing to couplets during stage 3 exercise at a maximum heart rate of 138 bpm. The comparatively low heart rate during nadolol treatment compared with the diagnostic heart rate at matched exercise intensities demonstrates the patient's compliance with β-blocker therapy and validates the treatment dose. The patient was then started on flecainide (50 mg twice daily; 2.7 mg/kg/day). In a follow-up stress test three weeks after starting flecainide, the patient was able to exercise to exhaustion with a peak heart rate during stage 3 exercise of 168 bpm and no ventricular ectopy (Fig. 1D).

### Patient-derived cells express lineage-specific markers

**Fig. 2.**
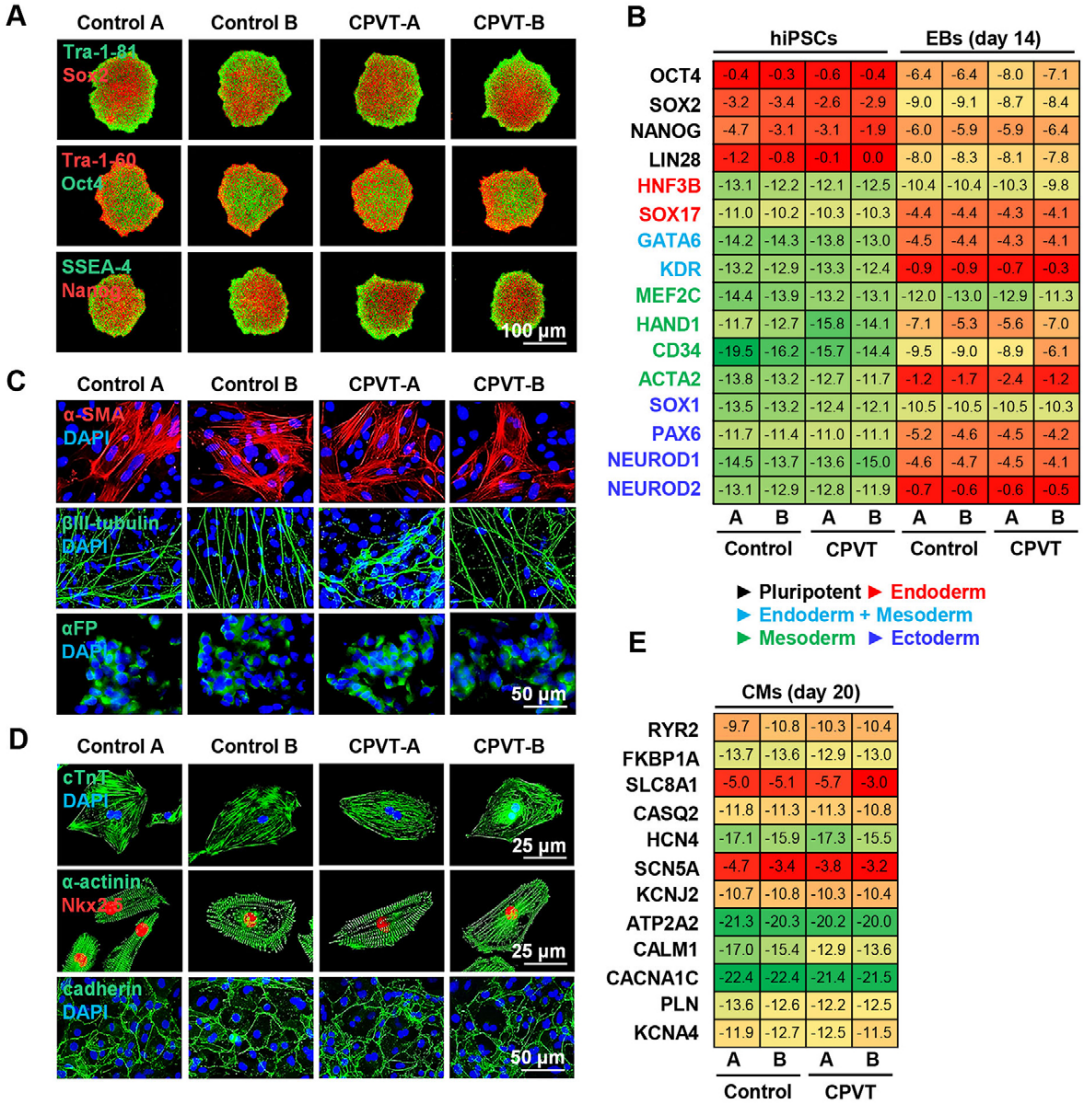
**Generation and differentiation of patient-derived induced pluripotent stem cells.** (A) Surface (Tra-1-81, Tra-1-60, SSEA-4) and intracellular (Sox2, Oct4, Nanog) expression of pluripotency markers in control and CPVT induced pluripotent stem cell (iPSC) lines. (B) Heat maps of pluripotency- and lineage-specific genes from iPSCs and embryoid bodies (EBs) illustrate mean gene expression levels presented as −ΔC_T_ values using a color gradient (green, low; yellow, medium; red, high). Differentiation of control and CPVT iPSCs into EBs results in downregulation of pluripotency genes and an increase in lineage-related genes. (C) EBs derived from control and CPVT iPSC lines express lineage-specific markers of mesoderm (α-smooth muscle actin, α-SMA), ectoderm (βIII-tubulin), and endoderm (α-fetal protein, αFP). (D) Cardiomyocytes differentiated from control and CPVT induced pluripotent stem cells were positive for cardiac markers cardiac troponin T (cTnT), α-actinin, Nkx2.5 and cadherin. (E) Heat maps illustrate mean expression levels as −ΔC_T_ values of 12 genes involved in cardiac excitation-contraction coupling using a color gradient (green, low; yellow, medium; red, high). Mean values are calculated from three samples per line per gene.

Two clonal iPSC lines were derived from the individual with CPVT (CPVT-A and CPVT-B), and two control lines (control A and control B) were derived from two unrelated healthy adult males with distinct genetic backgrounds. All four lines displayed typical iPSC colony morphology and normal 46, XY karyotypes (Fig. S1). Control and CPVT iPSCs were positive for pluripotency markers (Fig. 2A), and exhibited comparable expression of pluripotency genes as determined by qRT-PCR (Fig. 2B). Spontaneous differentiation of control and CPVT iPSC lines resulted in embryoid bodies (EBs) expressing lineage markers of endoderm (alpha fetal protein; αFP), mesoderm (α-smooth muscle actinin; α-SMA), and ectoderm (βIII-tubulin) (Fig. 2C). EB gene expression analysis revealed downregulation of pluripotency genes concomitant with upregulation of lineage-specific genes as determined by qRT-PCR (Fig. 2B). Directed differentiation of CPVT and control iPSCs resulted in beating CMs expressing cardiac markers cardiac troponin T (cTnT), α-actinin, Nkx2.5 and cadherin (Fig. 2D). qRT-PCR analysis was performed on 12 key genes involved in CM excitation-contraction coupling, and revealed similar levels of gene expression (Fig. 2E), consistent with existing reports (Jung et al., 2012; Kujala et al., 2012).

### CPVT iPSC-CMs exhibit altered Ca^2+^ homeostasis

**Fig. 3.**
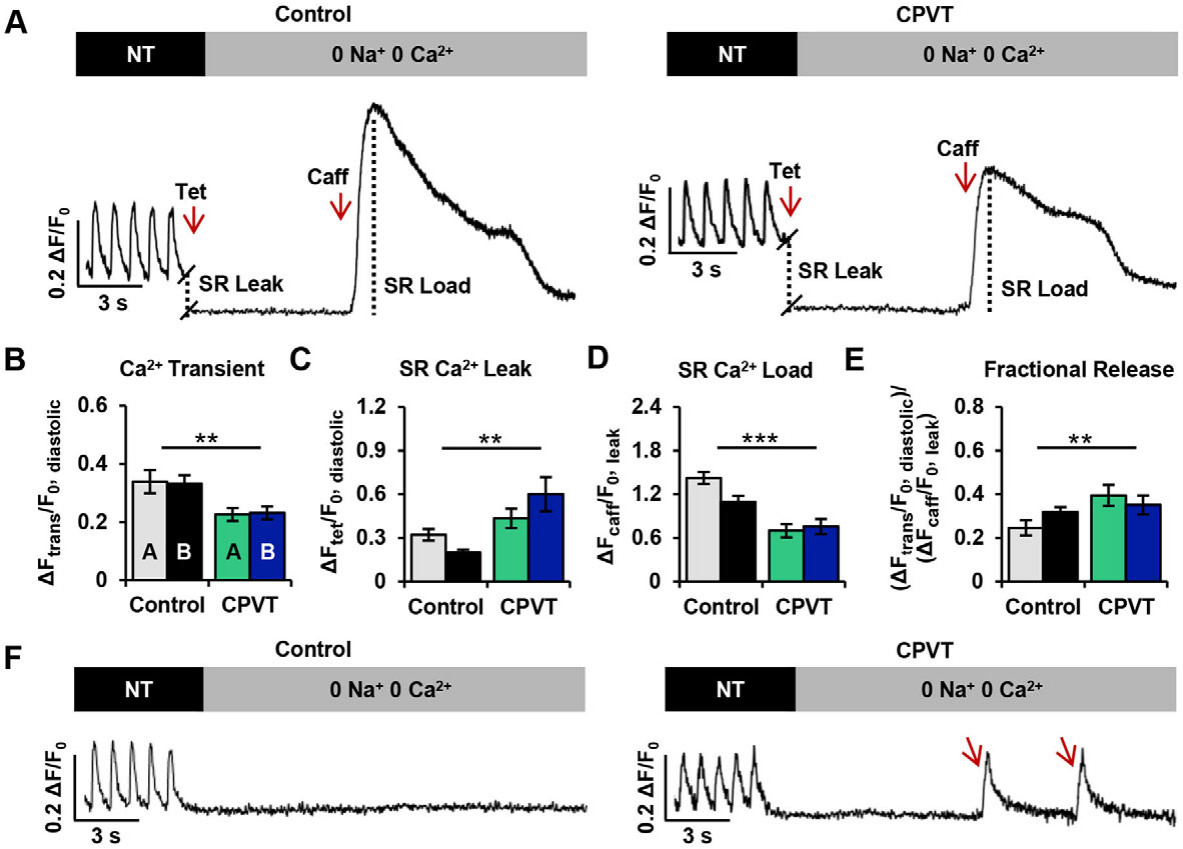
**CPVT iPSC-CMs exhibit altered Ca^2+^ homeostasis.** (A) Representative traces of cytosolic Ca^2+^ fluorescence in control and CPVT CMs paced at 1 Hz in NT solution and exposed to 0 Na^+^, 0 Ca^2+^ solutions containing tetracaine (Tet) and caffeine (Caff). (B-E) The protocol provided measurements of (B) Ca^2+^ transient amplitude (ΔF_trans_/F_0, diastolic_) during 1 Hz pacing, (C) sarcoplasmic reticulum (SR) Ca^2+^ leak (ΔF_tet_/F_0, diastolic_), (D) SR Ca^2+^ load (ΔF_caff_/F_0, leak_), and (E) fractional Ca^2+^ release (ΔF_trans_/F_0, diastolic_)/(ΔF_caff_/F_0, leak_). (F) Spontaneous Ca^2+^ oscillations (marked by red arrows) were present in CPVT, but not control cardiomyocytes during 0 Na^+^, 0 Ca^2+^ conditions. Data from cell lines CPVT-A (*n*=19) and CPVT-B (*n*=13) were combined and compared with the combined data from control A (*n*=11) and control B (*n*=19) for each variable. Bar graphs of control (*n*=30) and CPVT (*n*=32) CMs display mean±s.e.m., and significant differences are indicated as **P<0.01, ***P<0.001.

To test the hypothesis that normal Ca^2+^ homeostasis and contractility were affected in RyR2-L3741P CPVT iPSC-CMs, SR Ca^2+^ content and RyR2-mediated diastolic Ca^2+^ leak were assessed in CMs derived from both control (*n*=30) and CPVT (*n*=32) CMs. Representative traces illustrate the protocol for assessing the SR Ca^2+^ leak/load relationship (Fig. 3A). Cells were field-stimulated at 1 Hz in normal Tyrode (NT) solution for at least 20 s before being switched to 0 Na^+^, 0 Ca^2+^ Tyrode solution. Addition of tetracaine – a RyR2 inhibitor that occasions a global Ca^2+^ shift from the cytosol to the SR – induced a drop in diastolic Ca^2+^ fluorescence, the magnitude of which is an estimate of RyR2-mediated SR Ca^2+^ leak. Addition of a high concentration of caffeine – which evokes an exhaustive release of Ca^2+^ from the SR into the cytosol – induced a dramatic rise in Ca^2+^ fluorescence, an estimate of SR Ca^2+^ load. For more details on the interpretation of protocol data, refer to the Methods and materials section and Fig. S2.

No significant differences in Ca^2+^ homeostatic parameters were observed between control lines A and B or between CPVT lines A and B. Therefore, data from control A and control B and from CPVT-A and CPVT-B were combined for comparisons. The amplitudes of action-potential-induced Ca^2+^ transients during field stimulation (1 Hz) in NT solution were significantly ∼33% lower in CPVT CMs compared with control CMs (Fig. 3B). Additionally, the RyR2-mediated SR Ca^2+^ leak in CPVT CMs was significantly greater (∼35%) than in control CMs (Fig. 3C), and the caffeine-induced amplitudes of CPVT CMs were significantly lower (∼50%) compared with control, relating a reduced SR Ca^2+^ load (Fig. 3D). Fractional Ca^2+^ release was also significantly greater in CPVT CMs (∼61%) compared with control (Fig. 3E). During exposure to 0 Na^+^, 0 Ca^2+^ Tyrode solution, seven out of 32 CPVT CMs (∼22%) displayed extemporaneous Ca^2+^ oscillations, whereas all control CMs remained quiescent (Fig. 3F).

### Flecainide preferentially reduces spontaneous Ca^2+^ waves compared with β-blocker in CPVT iPSC-CMs during β-AR stimulation

**Fig. 4.**
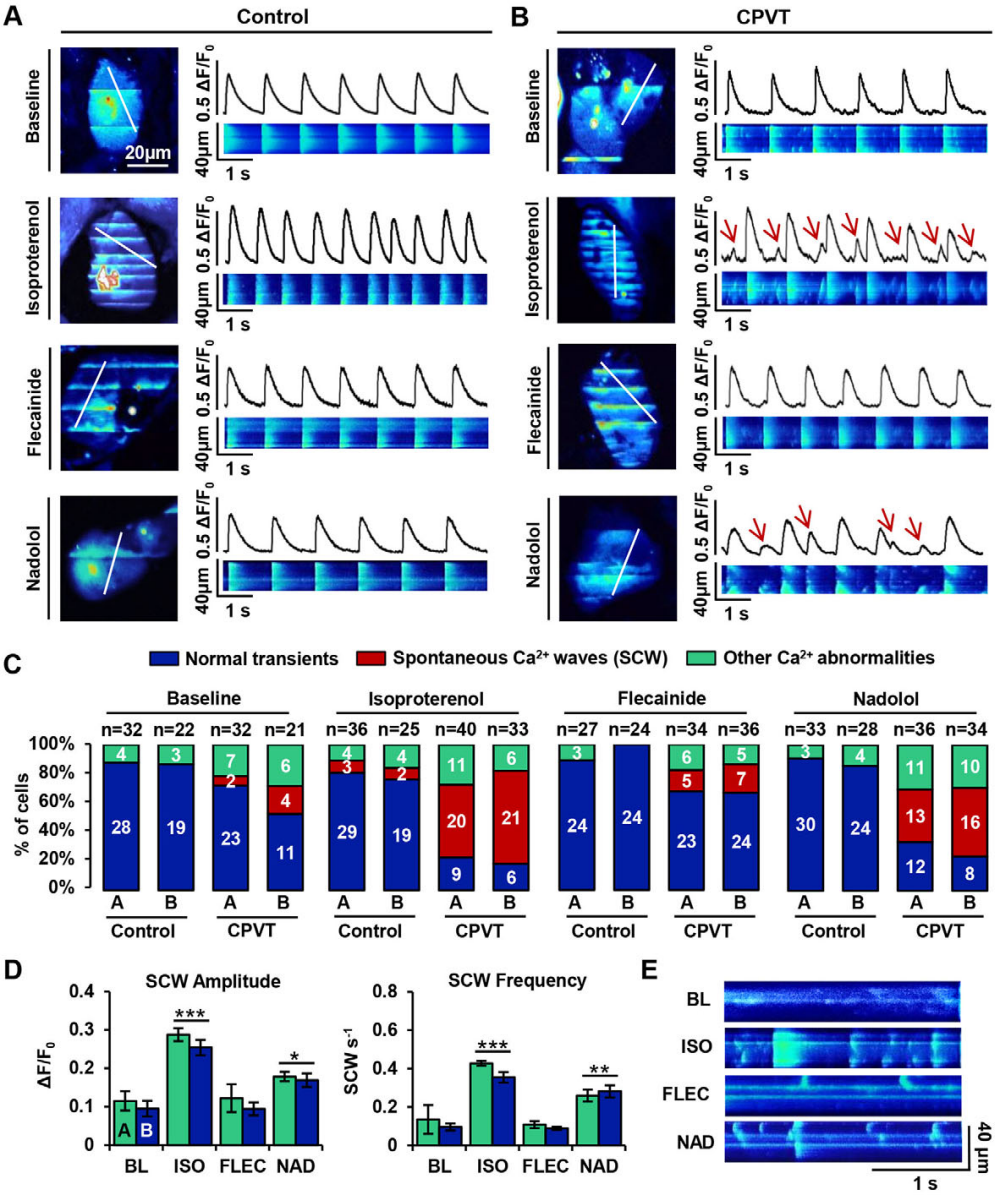
**Flecainide diminishes spontaneous Ca^2+^ waves in CPVT iPSC-CMs during β-AR stimulation.** (A,B) Representative confocal images and linear traces showing Ca^2+^ transients at baseline and during treatment with isoproterenol, flecainide and nadolol in (A) control and (B) CPVT CMs. Ca^2+^ transients and linear traces (*x*-time) correspond to the white line-scans drawn across the 2D images of the cell (*x*-*y*). Red arrows indicate incidences of spontaneous Ca^2+^ waves (SCW). (C) Stacked columns depict the number and percentage of cells exhibiting normal transients (blue), SCW (red) or other Ca^2+^ abnormalities (green) under each condition. Sample sizes (*n*) are denoted above each column for control A, control B, CPVT-A, and CPVT-B lines. (D) Of the cells exhibiting SCW, bar graphs represent the mean±s.e.m. amplitude (ΔF/F_0_) and frequency (waves/s) of waves for each CPVT cell line. For statistical comparisons between treatments, data from cell lines CPVT-A and CPVT-B were combined. Asterisks *P<0.05, **P<0.01, ***P<0.001 represent significant differences from baseline (BL; *n*_cell_=6, *n*_wave_=7) after treatment with isoproterenol (ISO; *n*_cell_=41, *n*_wave_=106), flecainide (FLEC; *n*_cell_=12, *n*_wave_=8), or nadolol (NAD; *n*_cell_=29, *n*_wave_=53). (E) Representative line-scans of cells displaying SCW during indicated treatments.

To determine whether *in vivo* responses of the individual with CPVT to β-blockade and flecainide could be mimicked *in vitro* using his iPSC-CMs, line-scan confocal cytosolic Ca^2+^ imaging was performed on spontaneously beating control (Fig. 4A) and CPVT (Fig. 4B) CMs at baseline, during β-AR stimulation with isoproterenol, and during additional treatment with either flecainide or nadolol. Recordings from this experiment were used for both Ca^2+^ transient and spark analyses.

In both control and CPVT lines, three main categories of Ca^2+^ transient were observed: normal transients, transients containing spontaneous Ca^2+^ waves (SCW), and transients exhibiting ‘other’, undefined abnormalities. Line-scans were categorized as ‘normal’ if the Ca^2+^ transients therein had mostly consistent amplitudes and beat periods, typical cardiac transient morphology (i.e. rapid upstroke and decay kinetics), and no notable instances of spontaneous Ca^2+^ release between transients (e.g. baseline transients in Fig. 4A,B). Line-scans were categorized as containing ‘SCW’ if they exhibited wavelets – oscillations of diastolic cytosolic [Ca^2+^] in specific regions of interests along a cell, or waves – whole-cell oscillations of diastolic cytosolic [Ca^2+^] (e.g. CPVT isoproterenol transients in Fig. 4B). For examples of ‘other,’ undefined Ca^2+^ transient abnormalities, refer to Fig. S3. The number of cells exhibiting each of these three transient classes during each condition was counted, and percentages were calculated for each cell line (Fig. 4C). At baseline, the vast majority of control cells beat regularly, with only a minor percentage of cells containing undefined transient abnormalities (∼13%) and none containing SCW. A greater incidence of undefined transient abnormalities was observed among CPVT cells (∼19%), with a few cells exhibiting SCW (∼11%). Isoproterenol treatment elicited a marked gain in Ca^2+^ cycling in both groups; however, the abundance of SCW in CPVT CMs precluded reliable quantification of the beating frequency. β-agonism induced SCW in ∼56% of CPVT CMs, but only minimally increased the percentage of control CMs with SCW to ∼8%. Upon treatment with flecainide or nadolol, control CMs exhibited negligible differences from baseline, and no incidents of SCW were observed. In CPVT CMs, flecainide and nadolol reduced the incidence of SCW by ∼70% and ∼27%, respectively.

No significant differences in SCW parameters were observed between CPVT lines A and B. Therefore, data from CPVT-A and CPVT-B were combined for comparisons between conditions. Compared with baseline, β-agonism dramatically increased both the amplitude (∼150%, *P*<0.0001) and frequency (∼260%, *P*<0.0001) of SCW (Fig. 4D). Compared with isoproterenol-treated CPVT CMs, flecainide-treated cells exhibited substantial reductions in SCW amplitude (∼58%, *P*<0.0001) and frequency (∼75%, *P*<0.0001). Although cells treated with nadolol during β-agonism also showed reductions in the amplitude (∼30%, *P*<0.0001) and frequency (∼30%, *P*<0.0001) of SCW, the drug was less effective than flecainide. Flecainide successfully rescued Ca^2+^ cycling during β-agonism, as there were no significant differences in SCW amplitude or frequency in flecainide-treated cells compared with baseline (Fig. 4D). In contrast, nadolol did not restore Ca^2+^ cycling to basal conditions, as SCW amplitude and frequency remained ∼70% (*P*<0.05) and ∼150% higher (*P*<0.01), respectively, in nadolol-treated cells compared with baseline (Fig. 4D). Confocal line-scans of cells displaying SCW under treatment conditions provide examples of this trend (Fig. 4E).

### Flecainide preferentially reduces Ca^2+^ spark abnormalities compared with β-blocker in CPVT iPSC-CMs during β-AR stimulation

**Fig. 5.**
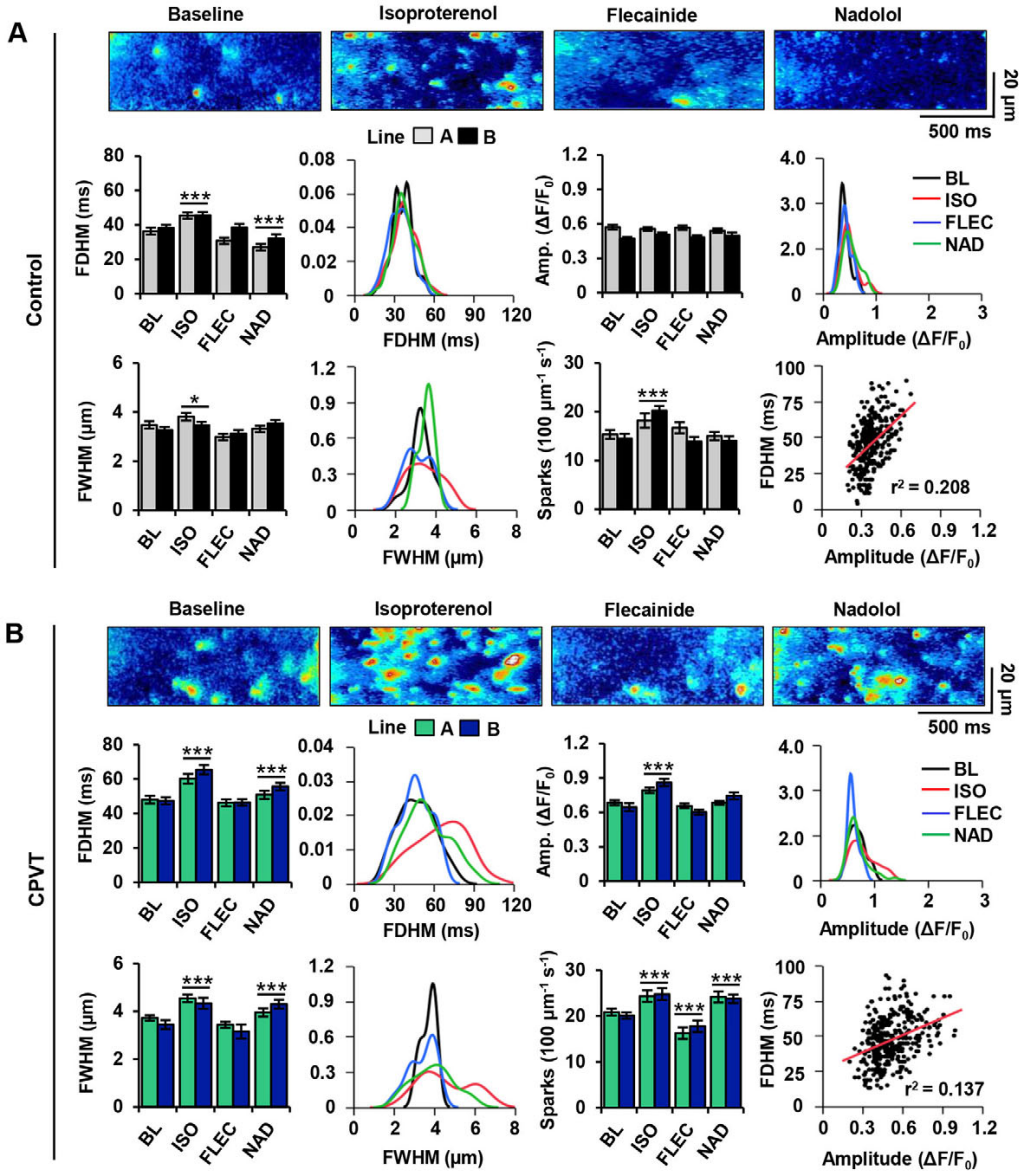
**Flecainide normalizes Ca^2+^ spark parameters in CPVT iPSC-CMs during β-AR stimulation.** (A) Spontaneous Ca^2+^ spark images and measurements in control CMs at baseline (BL; *n*_cell_=54, *n*_spark_=182), and after treatment with isoproterenol (ISO; *n*_cell_=61, *n*_spark_=272), flecainide (FLEC; *n*_cell_=51, *n*_spark_=162), or nadolol (NAD; *n*_cell_=61, *n*_spark_=158). (B) Spontaneous Ca^2+^ spark images and measurements in CPVT CMs at baseline (*n*_cell_=53, *n*_spark_=284), and after treatment with isoproterenol (*n*_cell_=73, *n*_spark_=264), flecainide (*n*_cell_=70, *n*_spark_=174), or nadolol (*n*_cell_=70, *n*_spark_=246). Bar graphs summarize mean±s.e.m. values for each cell line and each treatment: full duration at half maximum (FDHM), full width at half maximum (FWHM), amplitude (ΔF/F_0_), and spark frequency (100 µm^−1^ s^−1^). Data from cell lines control A and control B and from CPVT-A and CPVT-B were combined to generate the kernel density estimates and to compare data for each variable between treatments. Kernel density estimates depict the distributions (i.e. variability) of Ca^2+^ spark properties, and asterisks *P<0.05, ***P<0.001 represent significant differences from baseline. Correlations between amplitude and FDHM reveal only a weak linear relationship.

To investigate the ability of flecainide and nadolol to restore normal RyR2-mediated Ca^2+^ release at the elementary level in control and CPVT CMs, spontaneous Ca^2+^ sparks were analyzed across all treatment groups for several key parameters: full width at half maximum (FWHM), full duration at half maximum (FDHM), amplitude (ΔF/F_0_), and spark frequency (events per 100 µm per second) (Fig. 5A,B).

No significant differences in Ca^2+^ spark parameters were observed between control lines A and B or between CPVT lines A and B. Therefore, data from control A and control B and from CPVT-A and CPVT-B were combined for comparisons between conditions. At baseline, the mean FWHM and amplitude of Ca^2+^ sparks were comparable between control and CPVT CMs; however, FDHM and spark frequency were ∼27% and ∼33% higher, respectively, in CPVT CMs than in control. All spark properties in CPVT CMs were significantly increased by β-AR stimulation with isoproterenol, but amplitude did not increase in control CMs. In both control and CPVT CMs, flecainide treatment restored Ca^2+^ spark amplitude, FWHM and FDHM to pre-stimulation levels, showing no significant differences from baseline. Notably, spark frequency in CPVT CMs during flecainide treatment was significantly ∼10% lower than at baseline, representing levels similar to control CMs at baseline. With the exception of amplitude, nadolol treatment failed to restore spark parameters; spark frequency, FWHM, and FDHM remained significantly ∼13%, ∼14% and ∼12% higher, respectively, than at baseline.

In control CMs, Ca^2+^ sparks typically occurred randomly throughout the cell regardless of treatment (Fig. 5A). In contrast, sparks in CPVT CMs became highly recurrent during β-AR stimulation (Fig. 5B). The highly recurrent nature of Ca^2+^ releases was attenuated after flecainide, but persisted during nadolol treatment. Interestingly, the kernel density estimates of spark measurements in CPVT CMs reveal a greater spread in FWHM and FDHM distributions during isoproterenol and nadolol treatment than during flecainide treatment or at baseline (Fig. 5B). The kernel density estimates of spark measurements in control CMs were relatively consistent in spread across all conditions (Fig. 5A). Only a weak linear correlation was identified between FDHM and amplitude in control (r^2^=0.208) and CPVT CMs (r^2^=0.137) (Fig. 5A,B), indicating prolonged FDHM in CPVT CMs is not merely a function of increased amplitude.

### Flecainide mitigates electrical instability in CPVT iPSC-CMs during β-AR stimulation

To test the anti-arrhythmic potential of flecainide in CPVT CMs during β-AR stimulation, electrophysiological experiments were performed using microelectrode arrays (MEAs) as previously described (Navarrete et al., 2013). Extracellular field potential (FP) recordings were performed on spontaneously beating CPVT CMs at baseline, during β-AR stimulation with isoproterenol, and during additional treatment with flecainide.

**Fig. 6.**
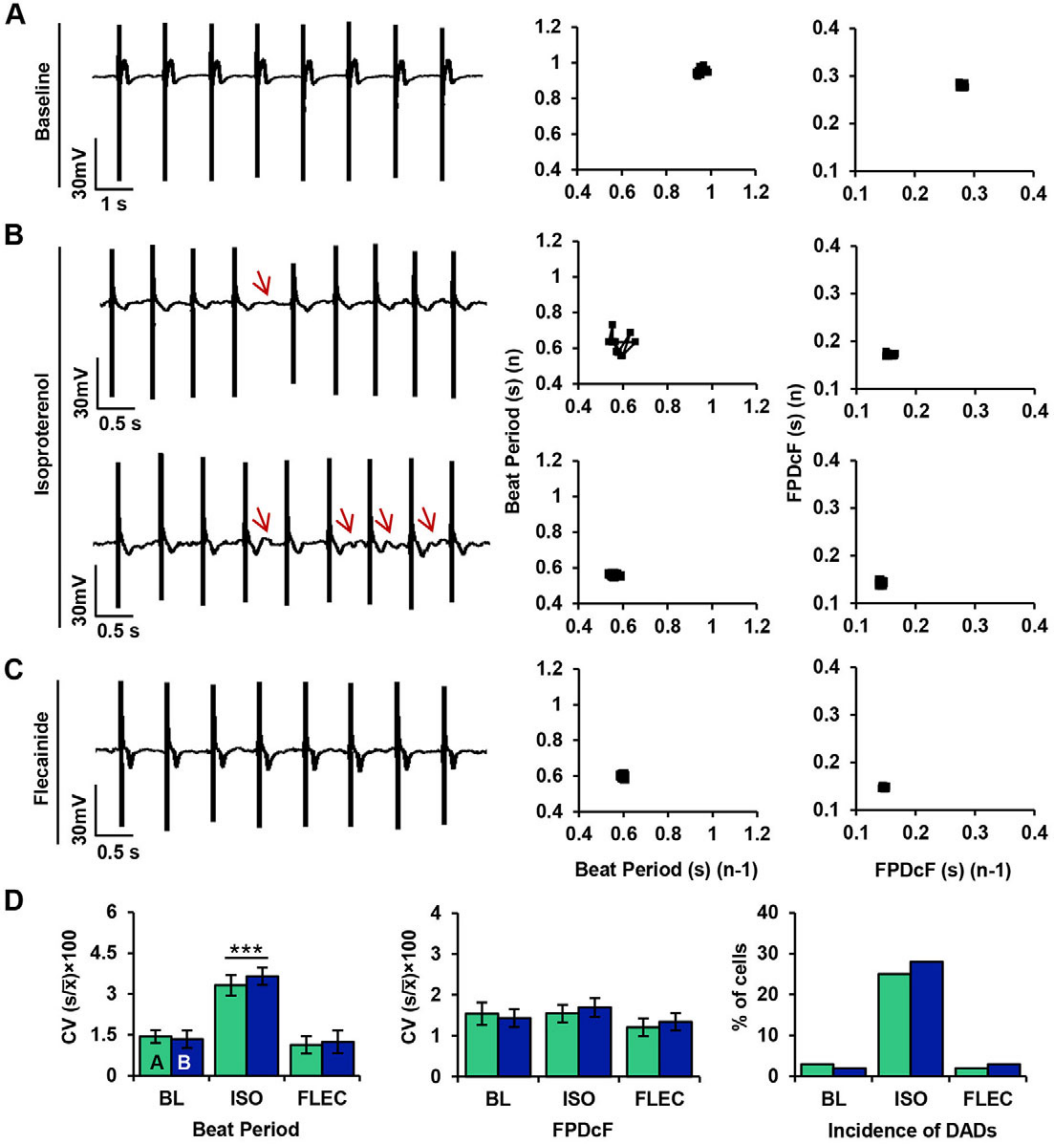
**Flecainide mitigates electrical instability in CPVT iPSC-CMs during β-AR stimulation.** (A-C) Representative microelectrode array recordings of extracellular field potentials in spontaneously beating CPVT CMs (A) at baseline, (B) during β-AR stimulation with isoproterenol, and (C) during additional treatment with flecainide. Poincaré plots for each trace illustrate variation in beat period and corrected field potential duration (FPDcF). During isoproterenol treatment, waveform abnormalities were observed (red arrows) in the form of beat period irregularities (top) or delayed afterdepolarizations (DADs) (bottom). (D) Bar charts display mean±s.e.m. of the coefficients of variation (CV) for beat period and FPDcF (left), and the percentage of cell aggregates displaying DADs (right) for both CPVT cell lines at baseline (BL; CPVT-A, *n*=12; CPVT-B, *n*=10), during β-AR stimulation with isoproterenol (ISO; CPVT-A, *n*=10; CPVT-B, *n*=11), and during flecainide treatment (FLEC; CPVT-A, *n*=9; CPVT-B, *n*=10). Significant differences from baseline are indicated as ***P<0.001.

Modified Poincaré plots of beat period and corrected field potential duration (FPDcF) in CPVT CMs show low beat-to-beat variability in both parameters at baseline (Fig. 6A). Isoproterenol treatment induced asymmetrical beating and increased variability in beat period (Fig. 6B). Furthermore, CPVT CMs exhibited sporadic occurrences of DADs during β-AR stimulation (Fig. 6B). Flecainide treatment restored beat period symmetry and eliminated DADs (Fig. 6C). Coefficients of variation (CV) were used to quantify rhythm irregularity for beat period and FPDcF. No significant differences in beat-to-beat variability were observed between CPVT lines A and B for either parameter; therefore, data from CPVT-A and CPVT-B were combined for comparisons between conditions. The variability in beat period was significantly higher (∼79%) during isoproterenol treatment compared with baseline (Fig. 6D). In contrast, no significant differences in beat-to-beat variability were observed during flecainide treatment compared with baseline (Fig. 6D). The percentage of cells displaying DADs during β-AR stimulation was dramatically higher (∼158%) than at baseline and during flecainide treatment (Fig. 6D).

### Flecainide restores Ca^2+^ homeostasis following β-AR stimulation in CPVT iPSC-CMs

**Fig. 7.**
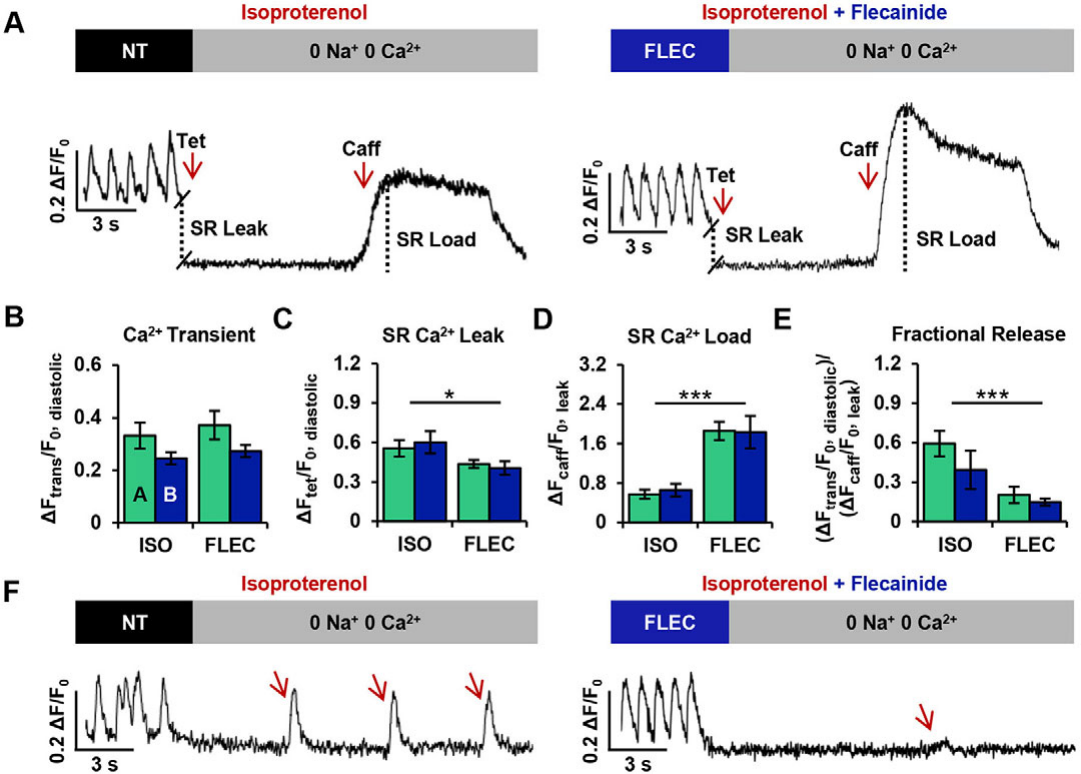
**Flecainide restores Ca^2+^ homeostasis following β-AR stimulation in CPVT iPSC-CMs.** (A) Representative traces of CPVT CMs pre-treated with isoproterenol and paced at 1 Hz in either NT solution or 10 µM flecainide. Cells were exposed to 0 Na^+^, 0 Ca^2+^ solutions containing tetracaine (Tet) and caffeine (Caff). (B-E) The protocol provided measurements of (B) Ca^2+^ transient amplitude (ΔF_trans_/F_0, diastolic_) during 1 Hz pacing, (C) sarcoplasmic reticulum (SR) Ca^2+^ leak (ΔF_tet_/F_0, diastolic_), (D) SR Ca^2+^ load (ΔF_caff_/F_0, leak_), and (E) fractional Ca^2+^ release (ΔF_trans_/F_0, diastolic_)/(ΔF_caff_/F_0, leak_). (F) Spontaneous Ca^2+^ oscillations in isoproterenol-treated cells were larger in amplitude and more frequent than in flecainide-treated CPVT CMs. Data from cell lines CPVT-A (*n*=7) and CPVT-B (*n*=7) during isoproterenol treatment were combined and compared with the combined data from CPVT-A (*n*=8) and CPVT-B (*n*=8) during flecainide treatment. Bar graphs of CPVT-A (*n*=14) and CPVT-B (*n*=16) CMs display mean±s.e.m., and significant differences are indicated as *P<0.05, ***P<0.001.

To gain insights into the mechanism by which flecainide minimizes spontaneous Ca^2+^ release and enhances electrical stability in CPVT CMs during β-AR stimulation, SR Ca^2+^ content and RyR2-mediated diastolic Ca^2+^ leak were assessed in CPVT CMs following isoproterenol exposure (*n*=14) and flecainide treatment (*n*=16). Representative traces illustrate the protocol for assessing the SR Ca^2+^ leak/load relationship for both treatment groups (Fig. 7A).

No significant differences in Ca^2+^ homeostatic parameters were observed between CPVT lines A and B. Therefore, data from CPVT-A and CPVT-B were combined for comparisons between conditions. The amplitudes of action-potential-induced Ca^2+^ transients during field stimulation (1 Hz) were not significantly different between treatment groups (Fig. 7B). The RyR2-mediated SR Ca^2+^ leak in the flecainide-treated group was significantly ∼21% lower than in the untreated group (Fig. 7C), and the caffeine-induced amplitudes of the flecainide-treated CPVT CMs were significantly ∼224% higher compared with untreated, relating a greater SR Ca^2+^ load (Fig. 7D). Fractional Ca^2+^ release was also significantly ∼66% lower in flecainide-treated cells compared with untreated (Fig. 7E). During exposure to 0 Na^+^, 0 Ca^2+^ Tyrode solution, extemporaneous Ca^2+^ oscillations were observed in five out of 14 (∼36%) of the untreated CPVT CMs, but only three out of 16 flecainide-treated CPVT CMs (∼19%). In addition to increased incidence, the oscillations in isoproterenol-treated cells were larger in amplitude and more frequent than in flecainide-treated CPVT CMs (Fig. 7F).

## DISCUSSION

**Fig. 8.**
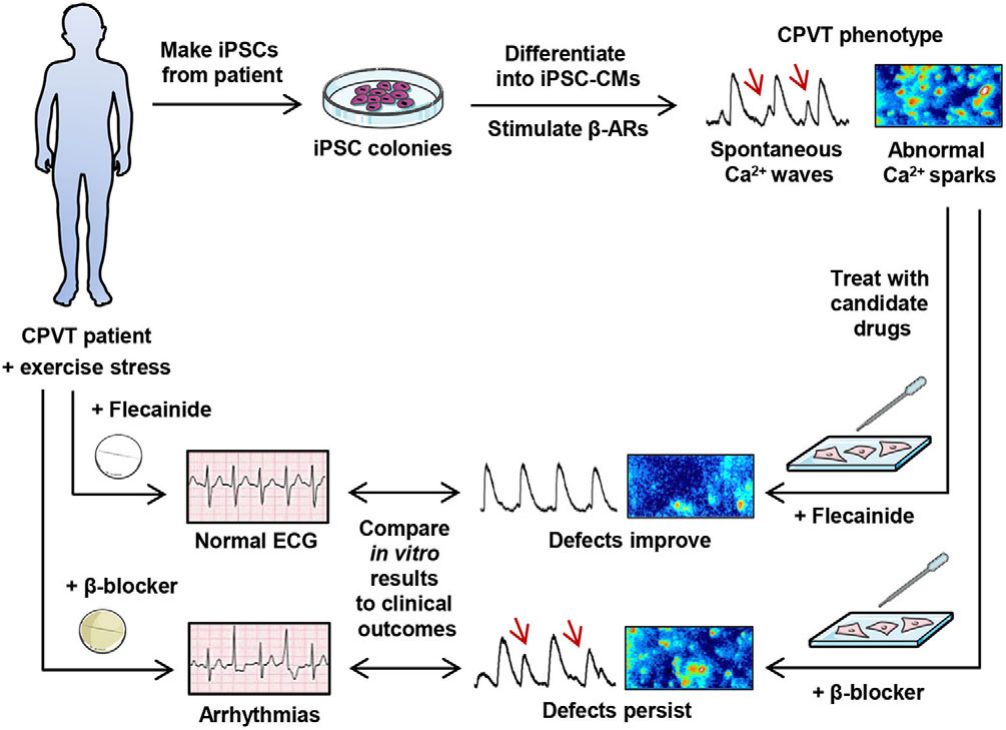
**Graphical summary.** CPVT patient-derived induced pluripotent stem cells (iPSCs) can be differentiated into CMs that exhibit the CPVT phenotype when stimulated with isoproterenol, a β-adrenergic receptor (β-AR) agonist. Following this, patient-specific drug responses to β-blocker and flecainide treatment can be observed *in vitro* that recapitulate clinical observations.

This study characterizes functional aspects of a novel RyR2 mutation, and presents evidence that a clinically observed therapeutic β-blocker response idiosyncrasy can be recapitulated *in vitro* using patient-specific iPSC-CMs (Fig. 8). As such, these results provide additional support for the use of patient-specific iPSC-CMs as an implement of precision medicine. Specifically, an individual with CPVT was identified whose persistent exercise-induced arrhythmias under tonic β-blockade with nadolol were abolished by treatment with flecainide. iPSC-CMs were generated from the individual and the cells challenged *in vitro* with the β-AR agonist isoproterenol to mimic catecholaminergic stimulation. β-AR agonism occasioned extemporal Ca^2+^ release in CPVT cells, manifesting as unduly large and prolonged diastolic Ca^2+^ sparks and SCW. The cells were then treated with either the β-blocker nadolol or flecainide, and both drugs were evaluated for efficacy. Pursuant to the patient's *in vivo* responses, β-blockade demonstrated negligible rescue of the isoproterenol-induced Ca^2+^ defects. In contrast, flecainide substantially improved Ca^2+^ handling in these cells by reducing the incidence, frequency and amplitude of SCW and restoring the size, frequency and duration of Ca^2+^ sparks to normalcy. Moreover, these results suggest that CM-specific factors are involved in patient-specific β-blocker responsiveness, as opposed to purely systemic (i.e. pharmacokinetic) differentials. It is possible that attenuated β-blocker efficacy is RyR2 mutation-specific, as KCNQ1 mutation-specific responses to β-blocker therapy have been observed in type I long QT syndrome, another inherited arrhythmogenic channelopathy (Barsheshet et al., 2012).

The state of basal Ca^2+^ homeostasis in these iPSC-CMs was assessed to gain insights into the CPVT patient's novel RyR2-L3741P mutation. As yet, two main types of CPVT-associated RyR2 mutations have been identified; gain-of-function (Jiang et al., 2002, 2005) and loss-of-function (Jiang et al., 2007; Zhao et al., 2015) mutations purportedly increase and decrease channel sensitivity to luminal Ca^2+^, respectively. Field-stimulated Ca^2+^ transient amplitudes were lower in CPVT compared with control CMs, indicating that Ca^2+^-induced Ca^2+^ release (CICR) competence is diminished in RyR2-L3741P CPVT CMs. Measurement of SR Ca^2+^ load and RyR2-mediated diastolic Ca^2+^ leak revealed mean SR Ca^2+^ load was significantly decreased in CPVT CMs, concomitant with significantly increased diastolic Ca^2+^ leak and enhanced fractional Ca^2+^ release. Moreover, in the absence of transsarcolemmal fluxes (0 Na^+^, 0 Ca^2+^), only CPVT CMs displayed Ca^2+^ oscillations. These oscillations ostensibly denote undue RyR2 channel activation during diastole, given that they were abolished upon RyR2 inhibition with tetracaine. As these properties have been associated with electrical instability and increased RyR2 sensitivity to luminal Ca^2+^ (Gonzalez et al., 2007; Iyer et al., 2007), we suspect that the RyR2-L3741P mutation is a gain-of-function mutation, although single-channel studies will be required to substantiate this claim.

Although cardiac arrhythmias are not exclusively Ca^2+^-dependent, intense or prolonged SR Ca^2+^ releases at the cellular level might be responsible for the distorted FP waveforms and premature depolarizations observed in our CPVT CMs. The emergence of arrhythmogenic SCW is contingent upon perturbations in the random activation, refractoriness and recruitment of RyR2 clusters (Weiss et al., 2011). Therefore, we theorize that these elements are disturbed during β-AR stimulation and are preferentially rescued by flecainide in our CPVT CMs. Firstly, Ca^2+^ sparks and other release events became significantly longer and highly recurrent in CPVT CMs during isoproterenol treatment compared with the brief and sporadic nature of Ca^2+^ sparks in controls, suggesting a disruption in the random activation of RyR2 channels and potentially elevated CICR gain (Zhang et al., 2013). Secondly, the higher frequency and amplitude of sparks and whole-cell SCW in CPVT CMs compared with controls is consistent with a previous study that reported accelerated Ca^2+^ release restitution in CPVT CMs attributed to abbreviated RyR2 refractoriness (Brunello et al., 2013). Thirdly, the increase in spatially large dyadic Ca^2+^ release in CPVT compared with control CMs during β-AR stimulation suggests enhanced recruitment of neighboring RyR2s. The greater mean and variability in Ca^2+^ spark duration observed in these CPVT CMs during β-AR stimulation compared with control could possibly be explained by impaired functional coordination (‘coupled gating’) of RyR2s (Marx et al., 2001), as mathematical models have demonstrated that even modest reductions in RyR2 coupling greatly increase the mean and variability of Ca^2+^ spark duration (Sobie et al., 2002). Notably, considerable reductions in both the mean and variability of Ca^2+^ spark duration were observed during flecainide, but not β-blocker, treatment.

Despite this, the exact mechanism of flecainide's salubrious effects in CPVT CMs remains a point of contention as highlighted in a recent review (Smith and MacQuaide, 2015). Given that flecainide significantly diminished RyR2-mediated SR Ca^2+^ leak and fractional release while increasing SR Ca^2+^ stores in CPVT CMs after β-agonism, one might be tempted to conclude that flecainide directly stabilizes RyR2, as has been previously reported (Hilliard et al., 2010; Galimberti and Knollmann, 2011). However, this premise has been brought into question by studies in wild-type animal CMs that suggest flecainide's inhibitory effects on the Na^+^ current (I_Na_) indirectly mitigate electrical instability through NCX (Sikkel et al., 2013) and Na^+^ channel-mediated rebalancing of intracellular Ca^2+^ homeostasis (Bannister et al., 2015). As we observed integrally disturbed Ca^2+^ homeostasis in RyR2-L3741P CPVT CMs, we speculate that flecainide's Ca^2+^-rebalancing mechanism of action could account for superior rescue of Ca^2+^-handling defects compared with the non-homeostatic effects of nadolol in these cells. Another possibility is that flecainide might additionally act by binding to one of the many cytoplasmic modulators of RyR2, such as FKBP12.6, calmodulin or S100A1 (Smith and MacQuaide, 2015).

Further patient-specific drug-response studies are needed that directly compare *in vitro* data from iPSC-CM experiments with clinical observations. Because iPSC-CMs express only fetal-like levels of Ca^2+^-buffering molecules (Yang et al., 2014), their relative immaturity could provide either an exaggerated or attenuated view of drug response differentials. Improved phenotypic data and access to patient genomic information will be important to elucidate if specific mutations or other genetic variants are predictive of β-blocker efficacy in CPVT, which could inform point-of-care treatment decisions. In conclusion, fundamental aspects of clinically observed patient-specific drug responses to β-blocker therapy in CPVT can be modeled *in vitro* using iPSC-CMs, and the efficacy of other drugs such as flecainide can be comparatively evaluated (Fig. 8). These findings advance proof-of-principle for patient-derived iPSC-CMs as an implement of precision medicine, and propound iPSC-CMs as a platform for exploring the molecular underpinnings of β-blocker treatment failure in CPVT.

## MATERIALS AND METHODS

### Patient identification and recruitment

Diagnosis of the CPVT-affected subject was determined after cardiac echocardiography, 24-h Holter monitoring, exercise stress testing (James et al., 1980), and electrocardiogram (ECG). Genotyping of the individual and his family was performed with the GeneDx genetic testing service using the CPVT sequencing panel. Control subjects were recruited voluntarily as healthy spouses of individuals affected with adult-onset neurological disorders. Control A in this study was a 59-year-old male and control B was a 62-year-old male. Both control individuals had no history of cardiac abnormalities or neurological disorders at the time of biopsy. Recent work has shown that reprogramming largely resets the biological age of donor somatic cells (Miller et al., 2013), obviating the need for age-matched controls. Informed consent was attained from all participants, and the study was approved as safe and ethical by the Emory University Institutional Review Board (IRB) in accordance with the principles expressed in the Declaration of Helsinki.

### Generation of cell lines, cell culture and cardiac differentiation

For CPVT lines, primary human dermal fibroblasts (hDFs) were reprogrammed into iPSC lines using integration-free episomal vectors as previously described (Okita et al., 2011). Low-passage (≤5) hDFs were used, as they exhibit enhanced reprogramming efficiency (Su et al., 2012). Approximately 5×10^5^ cells were transfected in 100 µl Amaxa nucleofection solution V (Lonza) with 1 µg each of Addgene plasmids 27077 (pCXLE-hOCT3/4-shp53-F), 27078 (pCXLE-hSK), and 27080 (pCXLE-hUL) using the Amaxa Nucleofector II/2b Device (program U-023). Transfected cells were cultured in fibroblast medium containing 10 µM ROCK inhibitor Y27632 (Stemgent) for one day. For the next 6 days, fibroblast medium was changed daily. On post-transfection day 6, the cells were dissociated and 1×10^5^ cells were re-plated onto irradiated mouse embryonic fibroblasts (MEF), and cultured in human pluripotent stem cell (hPSC) medium [KnockOut DMEM, 20% KnockOut SR, 2 mM L-glutamine, 0.1 mM non-essential amino acids, 0.1 mM β-mercaptoethanol (Sigma)] supplemented with 8 ng/ml human basic fibroblast growth factor (bFGF; Thermo Fisher) and Human iPS Reprogramming Boost Supplement II (EMD Millipore). All medium components were obtained from Thermo Fisher unless otherwise specified. The boost supplement was withdrawn on day 11. On post-transfection day 14, iPSC colonies were picked and re-plated at one colony per well. iPSCs were then cultured in ‘feeder-free’ conditions as previously described (Xu et al., 2001). Control lines were reprogrammed using the CytoTune iPS 2.0 Sendai Reprogramming Kit (Thermo Fisher) in accordance with manufacturer's instructions. For all lines, g-banded karyotyping was performed on live mitotic cell cultures (passages 10-20) using cytogenetics services (CPVT lines, Children's Hospital Oakland Research Institute; control lines, WiCell Research Institute). iPSCs were differentiated into cardiomyocytes using a growth-factor method and subsequently cultured in serum-free conditions as previously described (Jha et al., 2015).

### Immunocytochemical analysis

Adherent cells were rinsed with cold 1× PBS and fixed with 2% paraformaldehyde solution for 10-15 min at room temperature (RT), permeabilized with ice-cold 100% ethanol for 5 min, rinsed again with 1× PBS, and blocked overnight at 4°C with 5% normal goat serum (NGS; Thermo Fisher). Cells were incubated for 2 h at RT with primary antibodies, then rinsed three times with 1× PBS to remove excess antibody. Cells were incubated with fluorescently conjugated secondary antibodies for 1 h at RT in the dark. Cells were washed three more times with 1× PBS. Nuclear counterstaining was performed using Vectashield mounting media with DAPI (Vector Laboratories). For more information on the antibodies used, please refer to Tables S1 and S2. Cells were imaged with a phase contrast and fluorescence AxioVert A1 inverted microscope (Zeiss) equipped with AxioCam digital camera system (Zeiss). Images were exported using AxioVision LE (Zeiss) and merging was performed in Adobe Photoshop.

### Embryoid body pluripotency assay

The embryoid body (EB) assay for pluripotency was performed as previously described (Xu et al., 2001). Undifferentiated hiPSCs were harvested in clusters after incubation for 5 min at 37°C with collagenase IV (Life Technologies). Suspended colonies were then transferred to low-attachment plates in serum-containing differentiation medium [DMEM, 20% FBS, 1 mM L-glutamine, 0.1 mM β-mercaptoethanol (Sigma) and 1% non-essential amino acids]. All medium components were obtained from Thermo Fisher unless otherwise specified. Cell aggregates were maintained in suspension for 5 days before being transferred to 0.5% gelatin-coated plates. Attached EBs were maintained in differentiation medium for an additional 10 days to allow for random differentiation and the formation of outgrowths. On day 15, differentiated outgrowths were either harvested for RNA extraction or fixed with 2% paraformaldehyde for immunocytochemical analysis. Cultures were assessed for the presence of developmental germ layer markers via immunocytochemistry: α-smooth muscle actin (mesoderm), α-fetal protein (endoderm), and β-tubulin III (ectoderm). Gene expression levels for lineage-specific genes were assessed via qRT-PCR.

### Real-time polymerase chain reaction

Total RNA was extracted from iPSCs, 15-day-old EBs, and 20-day-old iPSC-CMs using Aurum total RNA mini kit (Bio-Rad). cDNAs were prepared from individual 1 µg RNA samples using the SuperScript VILO cDNA Synthesis Kit (Life Technologies), and incubations were performed using a C1000 touch thermal cycler (Bio-Rad). Gene expression levels were quantified using the 7500 Real-Time PCR System (Applied Biosystems). PCR amplifications were performed in skirted 96-well PCR plates (GeneMate) with iTaq Universal SYBR Green PCR Supermix (Bio-Rad). For each sample, mRNA levels were normalized to GAPDH mRNA levels. Data for each gene are presented in color-coded heatmaps of inverse ΔC_T_ values (corrected for GAPDH) as previously described (Schmittgen and Livak, 2008) on a scale of green (low) to medium (yellow) to red (high). Primer sequences were obtained from the NCI/NIH qPrimerDepot. For more information on the genes and primers selected, please refer to Tables S3 and S4.

### Assessment of Ca^2+^ homeostasis

Cardiomyocyte SR Ca^2+^ load and RyR2-mediated diastolic Ca^2+^ leakage were assayed using fluo-4 fluorescence and the Shannon–Bers technique (Shannon et al., 2002). Cardiomyocytes (25±5 days old) were incubated for 30 min at 37°C in cell culture medium containing 10 μM of the cytosolic Ca^2+^ dye fluo-4 AM (Life Technologies) to load the indicator into the cytosol. Following incubation, the indicator-containing medium was removed; cells were washed once, and incubated in cell culture medium for an additional 30 min at 37°C to allow for de-esterification of the indicator. Recordings were captured using an epifluorescence microscope (Olympus^®^ IX51) equipped with the IonOptix calcium and contractility system. Cells were bathed in 37°C NT solution (140 mM NaCl, 5.4 mM KCl, 0.53 mM MgCl_2_, 0.33 mM NaH_2_PO, 5 mM HEPES, 1.8 mM CaCl_2_, 10 mM glucose, pH 7.4 with NaOH) and field-stimulated at 1 Hz (30 V cm^−1^, 10 ms) for at least 20 s to bring the intracellular Ca^2+^ content to a steady state. Once achieved, stimulation was turned off, and the bathing superfusate was rapidly switched to a 0 Na^+^, 0 Ca^2+^ Tyrode buffer (140 mM LiCl, 5.4 mM KCl, 0.53 mM MgCl_2_, 5 mM HEPES, 10 mM glucose, 10 mM EGTA, pH 7.4 with LiOH) for 10 s to abolish transsarcolemmal Ca^2+^ fluxes through the Na^+^/Ca^2+^ exchanger (NCX). Tetracaine and caffeine solutions were added dropwise using a temperature-controlled needle directly above each cell. RyR2 channels were inhibited by adding a 1 mM tetracaine (0 Na^+^, 0 Ca^2+^) solution dropwise for ∼10 s. At this time, tetracaine was stopped, and SR Ca^2+^ stores were depleted by adding a 30 mM caffeine (0 Na^+^, 0 Ca^2+^) solution dropwise for ∼10 s. At this time, caffeine was stopped, and the bathing solution was rapidly switched from 0 Na^+^, 0 Ca^2+^ Tyrode buffer back to NT solution. Data files were exported and monotonic transient analysis was performed with IonWizard^®^ 6.5 software.

When cells were field stimulated at 1 Hz in NT solution, the action potential-induced Ca^2+^ transient amplitude was defined as ΔF_trans_/F_0, diastolic_, where ΔF_trans_ is the change in signal between the peak fluorescence and the minimum diastolic fluorescence (F_0, diastolic_). The tetracaine-induced drop in fluorescence was defined as ΔF_tet_/F_0, diastolic_, where ΔF_tet_ is the change in signal between the diastolic fluorescence preceding the addition of tetracaine (F_0, diastolic_), and the minimum fluorescence after tetracaine (F_0, leak_) under 0 Na^+^, 0 Ca^2+^ conditions. The caffeine-induced Ca^2+^ transient amplitude was defined as ΔF_caff_/F_0, leak_, where ΔF_caff_ is the difference between peak fluorescence after caffeine and the minimum fluorescence preceding the addition of caffeine (F_0, leak_) under 0 Na^+^, 0 Ca^2+^ conditions. Fractional Ca^2+^ release was defined as the ratio of the action potential-induced Ca^2+^ transient amplitude to the caffeine-induced Ca^2+^ transient amplitude (ΔF_trans_/F_0_, _diastolic_)/(ΔF_caff_/F_0, leak_). For a diagram that assists interpretation of these formulas, refer to Fig. S2.

### Imaging of intracellular Ca^2+^ dynamics and drug treatments

Spontaneously beating cardiomyocytes (30±2 days old) differentiated from control and CPVT iPSC lines were incubated with 10 μM of fluo-4 AM (30 min loading, 30 min de-esterification) at 37°C in culture medium, then transferred to an inverted laser confocal scanning microscope (Olympus FV1000) equipped with FluoView software (Olympus), where they were perfused with NT solution. For each pharmacological agent, cells were allowed to perfuse for 10 min with drug-containing NT before initiating recording. Fluo-4 was excited by the 488 nm line of an argon laser and emitted fluorescence was captured at >505 nm. Recordings of fluo-4 fluorescence were acquired in line-scan mode, where line-scans were preferentially positioned in the center of the cell (as opposed to the periphery) along the longitudinal axis (Guatimosim et al., 2011). Regions with densely accumulated fluo-4 were avoided, so as to exclude artifacts (e.g. endoplasmic reticulum, mitochondria, vesicles, etc.). Images were acquired in line-scan (X-T) mode at a sampling rate of ≥500 lines per second and a pixel size of 0.155 µm. Data was exported and analyzed with ClampFit^®^ 10.0 software (Molecular Devices).

As differentials in fluo-4 loading efficiency between cell lines can influence observed fluorescence intensities, loading and acquisition conditions were kept as consistent as possible, and all absolute fluorescence (F) measurements were normalized to inherent basal (i.e. background) fluorescence (F_0_). Estimates of intracellular Ca^2+^ are presented as changes in ΔF/F_0_, where ΔF=F−F_0_. Isoproterenol (100 nM), nadolol (10 µM), and flecainide (10 µM) dosages (Sigma) and incubation time (10 min) were initially selected according to previous literature reports (Audigane et al., 2009; Itzhaki et al., 2012; Mehta et al., 2014), but were validated by serial dilution in preliminary experiments based on their chronotropic effects (Fig. S4). The nadolol dosage was chosen as the concentration that returned the beating frequency approximately to baseline levels. The flecainide dosage was limited to 10 µM, as the two higher concentrations tested (25 µM and 50 µM) caused both control and CPVT cells to arrest beating.

### Ca^2+^ sparks analysis

Quantification of Ca^2+^ sparks was performed on line-scans using the SparkMaster plugin (Picht et al., 2007) for ImageJ (NIH). Regions for analysis were selected from the portions outside the action potential-induced transient. Fluorescence amplitudes were measured, normalized to basal fluorescence, and expressed as ΔF/F_0_. As recommended by the SparkMaster algorithm developers (Picht et al., 2007), a detection criteria threshold of 3.8 was selected in which the detection of events was 3.8 times the standard deviation of the background noise divided by the mean. Representative output images were generated using the ‘F/F_0_’ setting available in the plugin. *P*-values are reported for significant differences between treatment (e.g. isoproterenol) and baseline (i.e. no treatment), not for differences between CPVT and control CMs. Kernel density estimates of Ca^2+^ spark parameter distributions were generated for each condition using JMP from Statistical Analysis Software.

### Microelectrode array recordings and analysis

Microelectrode array (MEA) recordings were performed using the 64-channel Muse MEA system (Axion Biosystems). M64-GL (SU-8/Pt) MEA chambers (Axion Biosystems) were coated with matrigel (1:30 dilution) overnight before being seeded with 200 µl containing 6-12 CPVT iPSC-CM cell aggregates (day 20±5) per array. Spontaneously beating CPVT CMs were recorded at baseline and following 5 min incubation with 100 nM isoproterenol, and later 10 µM flecainide. Recording times were at least 1 min per sample per condition, and chambers were maintained at 37°C during recording. Analog field potential signals were acquired through a 0.1 Hz high-pass filter and a 2 kHz low-pass filter at a sampling frequency of 12.5 kHz. Data files were exported and analyzed using Axion Integrated Studio (AxIS) 2.3 software. Field potential parameters were analyzed, and waveforms were evaluated for the presence of DADs. Low-amplitude depolarizations occurring after completion of repolarization were considered DADs. Beat period (BP) was defined as the time in seconds between two successive depolarization spikes. Field potential duration (FPD) was defined as the time in milliseconds between a depolarization spike and the subsequent T-wave, and was corrected for beating rate using Fridericia's formula: 
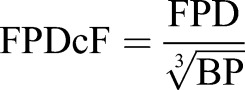
 (Nakamura et al., 2014). Modified Poincaré plots (Zhao et al., 2015) were generated by plotting the beat period time or FPDcF (converted to seconds) of beat *n* against that of beat *n*–1 for 60 beats. Coefficients of variation (CV) for beat period and FPDcF were calculated for each cell aggregate as the standard deviation (s.d.) of each parameter recorded in 60 s divided by the mean (x), multiplied by 100. Large CV values are indicative of beat irregularity and potential arrhythmia.

### Assessment of Ca^2+^ homeostasis in response to drug treatments

To determine the effects of β-agonism on SR Ca^2+^ leak and load, CPVT CMs were incubated for 10 min with 100 nM isoproterenol in NT solution at 37°C. After 10 min, the coverslips pre-treated with isoproterenol were perfused with NT solution for an additional 5 min before initiating the SR Ca^2+^ leak/load protocol. To assess the ability of flecainide to improve Ca^2+^ homeostasis in CPVT CMs during β-AR stimulation, cells were again pre-treated for 10 min with 100 nM isoproterenol, but were then perfused with 10 µM flecainide (rather than NT solution) for an additional 5 min before beginning the protocol.

### Statistical analysis

Across all data sets, significant differences between control lines A and B and between CPVT lines A and B were assessed by two-sample *t*-test. As no significant differences were observed, data from cell lines control A and control B and from CPVT-A and CPVT-B were combined, where necessary, for comparisons between conditions. For SR Ca^2+^ leak/load, significance was assessed by two-sample test. For Ca^2+^ spark analyses, spontaneous Ca^2+^ wave analyses, and MEA analyses, significance was assessed using one-way ANOVA, and post-hoc individual comparisons for each variable were performed using Tukey's HSD test.
